# Analysis of the Influence of Depression on the Occupational Performance of People Diagnosed with Multiple Sclerosis and Its Impact on Caregiver Burden

**DOI:** 10.1155/2024/7549306

**Published:** 2024-08-09

**Authors:** Sergio Rodríguez Menéndez, Marta Perez-de-Heredia-Torres, David Jaraba Berné, Manuel Menéndez González, Rosa Mª Martínez-Piédrola

**Affiliations:** ^1^King Juan Carlos University, Madrid, Spain; ^2^Department of Physiotherapy, Occupational Therapy, Rehabilitation and Physical Medicine, Faculty of Health Sciences, Rey Juan Carlos University, Madrid, Spain; ^3^Centre Hospitalari, Althaia, Spain; ^4^Department of Medicine, Neurology Service, Central Hospital of Asturias, Institute for Health Research of the Principality of Asturias., University of Oviedo, Oviedo, Spain

## Abstract

**Background:**

One of the most common symptoms in multiple sclerosis (MS) is depression, which causes disruption to daily participation. MS as a degenerative disease causes caregiver strain. The aim of this study is to analyze the influence of depression on the occupational performance of people with MS and to study whether these aspects influence caregiver strain.

**Materials and Methods:**

A descriptive cross-sectional observational study was carried out. A total of 124 people with a diagnosis of multiple sclerosis were assessed and administered the Beck Depression Inventory-II, the Canadian Occupational Performance Measure, and the Zarit Caregiver Burden Scale.

**Results:**

MS type influences performance, involvement, and caregiver burden. High levels of depression are associated with low levels of participation and performance. The types of MS with the highest caregiver burden are relapsing–remitting and secondary progressive.

**Conclusions:**

The type of MS negatively influences occupational performance. Depression and occupational performance are related to caregiver strain. The greater the depressive symptoms, the worse the performance, and the more caregiver strain.

## 1. Introduction

Multiple sclerosis (MS) is a chronic inflammatory and neurodegenerative demyelinating disease of the central nervous system (CNS), causing focal gray and white matter lesions and diffuse degeneration of the whole brain [1]. The disease can begin at any age, with onset around 20–40 years of age, most commonly affecting women [2]. An estimated 2.5 million people worldwide suffer from MS. In Europe, 700,000 people are affected. The areas with the highest prevalence correspond to high latitudes, and there has been an increase in prevalence in recent decades.

Also known as the disease of a 1,000 faces, its varied clinical manifestations affect motor, cognitive, sensory, and emotional aspects [3]. The uncertain course of the disease after diagnosis can lead to anxiety, stress, and depression, and they may feel like a burden on their support network. One of the most common symptoms in MS is depressive symptoms depression, with the prevalence of these symptoms being 2–4 times higher than in the general population [4]. Psychiatric symptoms can appear both as MS-specific symptoms, depending on the area and extent of the lesion, and as a comorbid pathology [5]. Nearly half of those diagnosed with MS have a clinically significant depressive episode in their lifetime. Diagnosis is difficult, but a multidisciplinary approach can improve quality of life and functional performance [6]. While all mental disorders affect the quality of life in all subjects, studies in recent years indicate that the presence of psychiatric disorders has a particularly negative effect on the patients' quality of life in MS [7]. Moreover, mental disorders in MS have also been associated with lower socioeconomic status due to job loss and increased social isolation, which in turn exacerbates depressive symptoms [8].

Mental disorders increase the perception of disability and increase caregiver burden [9]. A dependent person is defined as a person who, for reasons of age, illness, or disability, and linked to the loss of physical, sensory, mental, or intellectual autonomy, requires permanent care from another person or help to carry out their tasks care of another person or help in carrying out activities of daily living [10]. When we talk about the caregiver, we refer to the person responsible for assisting a dependent person in the basic and instrumental needs of daily living for most of the day [11], that is why it is needed to understand the patient's functionality in detail to address caregiver burden effectively. The level of caregiver strain in people with MS is higher than in other pathologies [12]. It is estimated that up to 58% of people diagnosed with MS are cared for by someone in their close family circle [13]. However, the precise mechanisms of this impact on quality of life and functionality have in MS not been described yet. In particular, the influence of depression on work performance MS remains unknown; and, in turn, it cannot established whether this potential influence is directly responsible for the impact of primary caregiver overload. Therefore, the present study aims to analyze the influence of depression on the work performance of people with MS and to study whether these aspects influence caregiver burden.

## 2. Materials and Methods

### 2.1. Study Design

A prospective cross-sectional descriptive observational study was conducted.

### 2.2. Participants

The entire sample was evaluated in the treatment centers of the associations that collaborated in this research: Asociación Viguesa de Esclerosis Múltiple (AVEMPO) and COCEMFE Gijón.

The inclusion criteria were to be diagnosed with MS, according to McDonald's criteria, in any of its stages, to be of legal age, and not to have suffered a flare-up in the last 12 months.

In addition, participants had to have a primary caregiver willing to participate in the study. Participants with a history of psychiatric illness before MS diagnosis were excluded.

### 2.3. Procedure

The administration of the tests was carried out in a place free of noise, with optimal lighting conditions, and with a space larger than 10 m^2^ to ensure a homogeneous environment. At the beginning of the assessment, a sociodemographic questionnaire was administered to obtain information on the independent variables (sex, age, type of MS, years of evolution, prediagnostic depressive symptomatology, and level of education). All tests were always administered by a single researcher. On the other hand, information regarding the level of disability obtained from the clinical reports of the participants was collected using the Expanded Disability Level Scale (EDSS).

Once consent was obtained, sociodemographic data were collected, and three assessment tests were administered in the following order:

1. Beck Depression Inventory-II composed of 21 items describing clinical symptoms, self-report type, which measures the severity of depressive symptoms; adapted from the Spanish population [3].
2. Canadian Occupational Performance Measure (COPM) establishes three areas of assessment (self-care, productivity, and leisure), in which the person identifies the most significant occupations in which he/she finds difficulties [14]. This tool gives us values related to occupational performance (COPM-R) and occupational performance satisfaction (COPM-S). It has been used in other studies in people diagnosed with MS in recent years [15].
3. Zarit Caregiver Burden Scale measures, with a numerical value, the degree of overload of caring for a dependent person. This overload (psychological, social, and physical) is assessed using 22 items scored from 0 to 4, the sum of which gives a value of between 0 and 88 points [16].

The average assessment time ranged from 45 to 60 min. All tests were administered by a single researcher.

### 2.4. Ethical Aspects

The study was approved by the ethics committee of the Universidad Rey Juan Carlos de Madrid, Spain (Registration number 0511201916019). All participants were informed in advance about the study, the tests, and the purpose, and the signature of their consent to participate was recorded.

### 2.5. Statistical Analysis

First, descriptive data were obtained for the entire sample, in terms of levels of functionality, and personal and sociodemographic variables. In them, the frequency of categorical variables and the mean or median, in some cases of continuous variables, can be observed. The existence of homogeneity of the sample was measured, verifying that not all the variables complied with normality, for which parametric and nonparametric tests were used. Second, the possible correlations between the different variables were analyzed using Spearman's nonparametric test and Pearson's parametric test. The statistical analysis was carried out using the statistical program IBM SPSS Statistics for Windows, Version 22.0 (© 2013, IBM SPSS Corp.).

### 2.6. Data Availability

Data associated with the article are not publicly available but are available from the corresponding author upon reasonable request.

## 3. Results

The final study sample consisted of 124 patients, of whom 42.7% were male and 57.3% female, with a mean age of 51.0 years (±10.8). According to the type of MS, 38.7% had secondary progressive MS, 16.9% had primary progressive MS, and 44.4% had relapsing–remitting MS. The mean number of years of disease evolution was 14.7 years (±10.6). All the participants were receiving pharmacological treatment for MS, with 39% of the total sample receiving treatment for depression. In relation to the caregivers, 82% were women while 18% were men, with an average age of 64.8 years (±6.8). Of these, 85% were family members while 15% were outsiders. In total, 11% of the sample of caregivers were on pharmacological treatment for depression.

Table 1 shows the results of the sociodemographic questionnaire, where we collect the sociodemographic variables that may influence depression.

Table 2 shows the descriptive data of the tests administered and their comparison between the different types of MS. In all scales, differences emerged depending on the type of MS. In the COPM-R and COPM-S scales, the score of patients with relapsing–remitting MS was significantly higher than that of patients with primary and secondary MS, with no statistically significant differences between patients with primary and secondary MS. On the other hand, the Zarit scale score of patients with relapsing–remitting MS was significantly lower than that of patients with primary and secondary MS, with no statistically significant differences between patients with primary and secondary MS. This means that caregivers of progressive types of MS present greater overload than those of relapsing–remitting type.

In the BECK scale, the score of patients with relapsing–remitting MS was significantly lower than that of patients with primary and secondary MS, and the score of patients with secondary MS was also lower than that of patients with primary MS, indicating that the type of MS that present greater depression are those with primary progressive, while those with less depressive symptomatology are those with relapsing–remitting.

With regard to COPM (Table 3), in the COPM-R dimension, the variables with a significant effect were secondary and remitting MS (patients with these types of MS have lower COPM-R than patients with primary MS, respectively), years of evolution (as years of evolution increase, COPM-R score decreases), and BECK scale scores (high BECK values are associated with low COPM-R levels). In the COPM-S dimension, the variables with a significant effect were secondary and remitting MS (patients with these types of MS have lower COPM-S than patients with primary MS, respectively), and BECK (high BECK values are associated with low COPM-S).

In the ZARIT scale (Table 4), the variable with a significant effect was secondary and remitting MS (patients with these types of MS have a lower ZARIT than patients with primary progressive MS).

## 4. Discussion

The present study aimed to analyze the influence of depression on occupational performance in people with MS and to study whether these aspects influence caregiver strain.

In our study population, the results obtained show a greater severity of depressive symptoms in women compared to men, as in other studies conducted in MS patients and in the general population [4]. Interestingly, although women have a lower prognosis in terms of long-term disability, they have a higher mortality ratio [17], so the results obtained may reflect fear about the course of the disease and not just levels of disability. It is also observed that the score is higher in patients with primary progressive MS than in those affected by secondary progressive MS, and in these two groups, it is significantly higher than in patients with relapsing–remitting MS. In this study, we found a relationship between occupational performance and mood, indicating that the higher the score on the Beck scale, the lower the self-perception of performance.

No previous studies have been found that relate these variables in people with MS, although studies in older adults point to the same trend of results [18]. Regarding the correlation between depression scores and caregiver burden, our data indicate that the greater the severity of depressive symptoms, the greater the caregiver burden. These results may contribute to improved treatments and bring us closer to a more ecological rehabilitative approach. Previous studies in the MS population have focused on the caregiver's depressive symptoms [12], although other studies indicate that this burden increases when users with the disease begin to present nonmotor [13]. Therefore, according to the results obtained, both occupational performance and caregiver burden are affected by the increase in depressive symptoms, being significantly higher in the relapsing–remitting type of MS. These results show some differences with previous studies, where a higher number of depressive symptoms were observed in the sample with secondary progressive MS [19].

As limitations of the study, it should be noted that the study sample corresponds to the same geographical area and that the age range of the sample was very wide. It would be interesting for future research studies to take into account the type of medication taken, both at a general level as disease modifiers and specifically for depression. More importantly, the best method to diagnose mental disorders is through a structured interview. Depression inventory is not a diagnostic tool as such. It would also be interesting to study whether caregiver burden is related to depression in caregivers, and how all these factors interact to impact the quality of life in patients and caregivers. Further and thorough studies—based on other instruments—are needed to better understand the interaction of these variables and analyze possible differences according to socioeconomic and cultural factors.

## 5. Conclusions

The results of this work allow us to broaden our understanding of the influence of depression on work performance and caregiver strain. Depressive symptoms have a high prevalence in people diagnosed with MS, even more so in women. The type of MS is also a factor to be taken into account in terms of depression and it has a negative influence on work performance. People with remitting MS are those with less depressive symptoms and better self-perception of occupational performance. Depression and occupational performance are related to caregiver strain. The more depressive symptoms, the worse performance, and the greater caregiver strain. Caregiver overload is present in all three types of MS.

Taking these results into consideration can help us to design the lines of intervention that promote the participation of people with MS in their habits and routines and tackle caregiver burden more effectively.

## Figures and Tables

**Table 1:** Sociodemographic variables.

	Men (%)	Women (%)
Mean age	52 (43%)	72 (57%)
40–50	3 (2%)	9 (7%)
50–60	35 (29%)	43 (35%)
>60	14 (12%)	20 (15%)
Education		
Less than college	9 (7%)	15 (12%)
College	39 (31%)	43 (35%)
University	4 (3%)	14 (10%)
Prediagnosis depression	4 (3%)	9 (7%)
EDSS		
0.00–4.00	32 (26%)	40 (32%)
4.00–7.50	17 (14%)	30 (24%)
7.50–10.00	3 (3%)	2 (1%)

**Table 2:** Descriptive and comparative scales according to type of MS.

Test	MS type						ANOVA		*η* ^2^
	Secondary		Primary		Relapsing		*F* (2, 121)	*p*-Value	
COPM-S	5.17 (2.19)	A	3.62 (2.06)	b	8.56 (1.83)	c	60.71	**<**0.001	0.50
COPM-R	5.98 (2.33)	B	4.76 (2.66)	b	9.16 (1.77)	a	43.63	<0.001	0.42
ZARIT	56.98 (13.16)	B	60.19 (9.07)	b	10.56 (11.78)	a	241.18	<0.001	0.80
BECK	33.54 (18.43)	A	45.33 (10.05)	b	15.53 (10.97)	c	40.31	<0.001	0.40

a—b—c, two-to-two comparisons. Between two types of MS if no letter is shared, it indicates statistically significant differences (Bonferroni correction). *η*^2^, partial eta-squared (effect size).

**Table 3:** COPM-scale regression models.

	COPM-R				COPM-S			
	*B* (ET)	*β*	*t*	*p*-Value	*B* (ET)	*β*	*t*	*p*-Value
Sex (women vs. men)	−0.61 (0.38)	−0.11	−1.63	0.106	−0.51 (0.37)	−0.09	−1.36	0.176
Age	0.04 (0.02)	0.13	1.47	0.143	0.01 (0.02)	0.04	0.44	0.664
MS type								
Secondary vs. primary	−1.50 (0.54)	−0.26	−2.76	**0.007**	−2.13 (0.54)	−0.37	−3.99	**<0.001**
Remitting vs. primary	−2.18 (0.70)	−0.29	−3.13	**0.002**	−3.18 (0.69)	−0.42	−4.64	**<0.001**
Years of evolution	−0.07 (0.03)	−0.23	−2.68	**0.008**	−0.05 (0.03)	−0.15	−1.73	0.086
Beck	−0.06 (0.01)	−0.38	−4.59	**<0.001**	−0.05 (0.01)	−0.29	−3.64	**<0.001**
*R* ^2^ (%)	54				55.8			
Model	*F* (12, 110) = 12.96, *p* < 0.001				*F* (12, 110) = 13.84, *p* < 0.001			

*B*, regression coefficient; ET, standard error; *t*, size of the difference in relation to the variation in the sample data. Significant result are maked in bold type.

**Table 4:** ZARIT scale regression model.

	*B* (ET)	*β*	*T*	*p*-Value
Sex (women vs. men)	−0.63 (2.39)	−0.01	−0.26	0.793
Age	−0.20 (0.15)	−0.08	−1.32	0.19
MS type				
Secondary vs. primary	46.21 (3.44)	0.86	13.43	<0.001
Remitting vs. primary	48.56 (4.42)	0.69	10.99	<0.001
Age of evolution	0.19 (0.17)	0.06	1.09	0.277
Beck	0.02 (0.08)	0.01	0.22	0.828
*R* ^2^ (%)	79			
Model	*F* (12, 110) = 39.24, *p* < 0.001			

*B*, regression coefficient; ET, standard error; *t*, size of the difference in relation to the variation in the sample data.

## Data Availability

The data of the present study are stored at the Universidad Rey Juan Carlos, Alcorcón campus, where only the principal investigator has access by means of a password and encrypted access.
